# FALL INJURY CHARACTERISTICS ASSOCIATED WITH EMERGENCY DEPARTMENT VISITS AND HOSPITALIZATIONS AMONG OLDER ADULTS

**DOI:** 10.1093/geroni/igz038.1836

**Published:** 2019-11-08

**Authors:** Namkee G Choi, Diana M DiNitto, Mark E Kunik

**Affiliations:** 1 University of Texas at Austin, Austin, Texas, United States; 2 Baylor College of Medicine, Houston, Texas, United States

## Abstract

Fall injuries and related healthcare use among older adults are increasing in the US. Based on the 2013-2017 US National Health Interview Survey public use data, this study examined fall injury characteristics that are associated with emergency department (ED) visits and hospitalizations among those aged ≥60 years who received medical attention for their fall injuries within a 91-day reference period (N=1,840). Our findings show that nearly a third of these older adults received care from emergency medical services (EMS), presumably for a “lift assist” to get off the floor and/or for ED or hospital transport; a little more than one-third had an ED visit only; and a little less than a fifth had an overnight hospital stay. Multivariable analysis showed that hip and head injuries, face injuries, and broken bones/fractures (from any type of injury) were likelier causes of hospitalization than injuries to other parts of the body. Fall injuries sustained inside the home, falls from loss of balance/dizziness, and living alone were also more likely to result in hospitalization, while fall injuries that occurred away from home and those with lung disease and memory problems were associated with higher risk of ED use only. These healthcare use data indicate the significant toll that fall injuries exact upon older adults and healthcare system. Fall prevention programs should target risk factors that are specific to serious injuries and be made more accessible. Strategies for implementing scalable, adaptable, and measurable fall prevention models by EMS providers and ED staff are also needed.

